# Comparison of Scanpy-based algorithms to remove the batch effect from single-cell RNA-seq data

**DOI:** 10.1186/s13619-020-00041-9

**Published:** 2020-07-06

**Authors:** Jiaqi Li, Chengxuan Yu, Lifeng Ma, Jingjing Wang, Guoji Guo

**Affiliations:** 1 Center for Stem Cell and Regenerative Medicine, The First Affiliated Hospital, Zhejiang University School of Medicine, Hangzhou, 310058 China; 2 Zhejiang Provincial Key Lab for Tissue Engineering and Regenerative Medicine, Dr. Li Dak Sum & Yip Yio Chin Center for Stem Cell and Regenerative Medicine, Hangzhou, 310058 China; 3 Bone Marrow Transplantation Center, The First Affiliated Hospital, Zhejiang University School of Medicine, Hangzhou, 310009 China; 4 Institute of Hematology, Zhejiang University, Hangzhou, 310058 China; 5 Stem Cell Institute, Zhejiang University, Hangzhou, 310058 China

## Abstract

With the development of single-cell RNA sequencing (scRNA-seq) technology, analysts need to integrate hundreds of thousands of cells with multiple experimental batches. It is becoming increasingly difficult for users to select the best integration methods to remove batch effects. Here, we compared the advantages and limitations of four commonly used Scanpy-based batch-correction methods using two representative and large-scale scRNA-seq datasets. We quantitatively evaluated batch-correction performance and efficiency. Furthermore, we discussed the performance differences among the evaluated methods at the algorithm level.

## Background

Single-cell RNA sequencing (scRNA-seq) technology provides significant support and assistance for researchers to explore intercellular heterogeneity and gain insight into biological processes (Hwang et al. 2018; Shalek et al. 2014; Zeng and Dai 2019). As the cost of sequencing has decreased and large-scale cell atlas projects have been established, researchers are facing the challenge of processing single-cell sequencing data for even millions of cells (Macosko et al. 2015; Gierahn et al. 2017; Klein et al. 2015; Han et al. 2018; Tabula Muris, and Overall c, Logistical c, Organ c, processing, Library p, sequencing, computational data a, Cell type a, Writing g 2018). Such large-scale sequencing data usually require the integration of multiple experiments, which may include data generated by different laboratories using different cell isolation methods, RNA capture and processing methods, library preparation methods, and sequencing platforms. However, scRNA-seq captures both biological and technical variations, the latter of which is difficult to distinguish from the former when integrating multiple scRNA-seq datasets (Stuart and Satija 2019; Tung et al. 2017). Simply integrating the digital gene expression (DGE) matrix across different batches may introduce additional nonbiological bias and noise to the gene expression counts. Generally, the batch effect is used to describe nonbiological experimental variation caused by sampling distinct experimentally or technologically derived batches (Johnson et al. 2007a). These kinds of technical biases and systematic noises may mask the biological differences between cells (Wang et al. 2019). Therefore, batch correction is one of the key steps in scRNA-seq dataset integration for removing the batch effect and preserve biological variation. It is necessary to select the appropriate method to correct batch effects before data integration and downstream analysis. The Seurat v3 package in R is a very powerful data-analyzing tool for scRNA-seq data, which includes integration and batch-effect correction for multiple experiments based on the “anchors” strategy (Stuart et al. 2019). However, Seurat usually takes a long time to integrate and process a relatively large dataset. Scanpy is a python implementation of a single-cell RNA sequence analysis package inspired by Seurat (Wolf et al. 2018). There are many batch-correction methods based on the Scanpy platform with advantages over Seurat in terms of processing efficiency and running speed. This means that under the same hardware conditions, it takes less time for tools on the Scanpy platform to process scRNA-seq data.

In this study, we selected four commonly used and relatively well-developed Scanpy-based algorithms: Regress_Out (Wolf et al. 2018), ComBat (Johnson et al. 2007b), Scanorama (Hie et al. 2019), and MNN_Correct (Haghverdi et al. 2018). The Regress_Out algorithm uses simple linear regression to regress out unwanted sources of variation. The ComBat method uses the empirical Bayes framework in linear regression to achieve statistical power with information across genes. Instead of using linear regression, the MNN_Correct algorithm detects mutual nearest neighbors (MNNs) in the high-dimensional expression space for batch correction. Similarly, Scanorama uses randomized singular value decomposition (SVD) to compress the gene expression profiles into a low-dimensional embedding and further searches the MNNs for batch correction. In this study, we used two representative mouse scRNA-seq resources to demonstrate their performances and characteristics in correcting batch effects. First, we used lung datasets to perform intratissue evaluation. Next, we used kidney datasets to validate the intratissue performance. Finally, we performed evaluations across different tissues. We used two quantitative metrics as well as performance time to evaluate the batch-correction performance. Findings from this work will not only inform current discussions on the integration of multiple scRNA-seq datasets but also provide some suggestions for the future development of batch-correction methods.

## Methods

### Single-cell RNA-seq datasets

To test the four aforementioned batch-correction methods, we used two representative and publicly available mouse scRNA-seq resources: the Mouse Cell Atlas (MCA) (Han et al. 2018) using the Microwell-seq technique and the Tabula Muris (TM) (Tabula Muris, and Overall c, Logistical c, Organ c, processing, Library p, sequencing, computational data a, Cell type a, Writing g 2018) using 10x Genomics. From the MCA dataset, we selected 26 batches across 3 developmental stages from 23 tissues, including the adrenal glands, bone marrow, brain, calvaria, heart, kidney, liver, lung, male gonad, muscle, ribs, omentum, ovary, pancreas, peripheral blood, placenta, pleura, prostate, small intestine, spleen, stomach, testis and uterus. For the TM dataset, we selected 28 batches from 12 tissues, including the bladder, heart and aorta, kidney, limb muscle, liver, lung, mammary glands, marrow, spleen, thymus, tongue, and trachea. We filtered cells with less than 500 UMI (Unique Molecular Index) in both the MCA and TM datasets. Compared with that of the 10x Genomics in TM, the sequencing depth of the MCA dataset is slightly shallower, but considerable cell flux can be obtained at a lower cost.

Selected data sets were organized to represent the common single-cell data integration scenarios of the intratissue and across-tissue batch corrections. Lung data from the MCA and TM datasets were selected to represent the intratissue integration scenario. The MCA lung data contain three experimental batches, and the TM lung data contain four experimental batches. Moreover, we repeated this scenario using the MCA and TM kidney data to validate the performance of the four batch-correction methods. For the across-tissue batch correction scenario, we evaluated multiple experimental batch data, including the 26 experimental batches of the 23 tissues from the MCA database and the 28 experimental batches of the 12 tissues from the TM database.

### Comparison of the four batch-effect correction tools

Scanpy is a python implementation of a single-cell RNA sequence analysis package inspired by the Seurat package in R. Using the standard Scanpy workflow as a baseline, we tested and compared four batch-effect correction tools, including Regress_Out, ComBat, Scanorama, and MNN_Correct.

In the standard Scanpy pipeline, we first filtered cells with fewer than 200 genes and genes with fewer than 3 cells as a simple quality control. After performing normalization to 1e4 counts per cell and calculating the base-10 logarithm, we selected highly variable genes using the standard Scanpy filter_genes_dispersion function with the default parameters. The unwanted variations of ‘n_counts’ and ‘percent_mito’ were regressed out before we performed the standard batch-correction function of each of the four batch-correction methods.

The four algorithms, Regress_Out, ComBat, Scanorama and MNN_Correct, were run using the Scanpy sc.pp.regress_out, sc.pp.combat, scanorama.correct and sc.external.pp.mnn_correc functions, respectively, to remove the batch variations.

Then, we calculated the principle components of the batch-corrected gene expression matrix and uniformly selected the top 45 PCs for downstream analysis. A shared nearest neighbor (SNN) graph was constructed using the pp.neighbor function with 15 neighbors, and the t-SNE embedding space was calculated using 30 perplexities to visualize the result. Finally, the Louvain method with a fixed resolution of 0.6 was used to cluster the single cells into specific cell types to compare the performance of the four batch-correction algorithms on unsupervised clustering.

### Evaluation of batch-correction performance

To compare the clustering results of the four batch-correction methods, we employed two quantitative metrics to evaluate the batch-correction performance: the k-nearest neighbor batch-effect test (kBET) and the average silhouette width (Buttner et al. 2019; Rousseeuw 1987).

Using SVD-based dimension reduction, kBET computes the local batch label distribution of the detected k-nearest neighbors and randomly selects 10% of the cells to test the local batch label distribution against the global distribution. The null hypothesis of all batches being well mixed is rejected if the local distribution is different from the global distribution. After PCA dimension reduction, we input the top 20 PCs from the batch-corrected gene expression matrix to the kBET function. The results of the kBET are impacted by the selection of a predefined number of k nearest neighbors. We used diverse k input values to run the kBET function, and the mean values of all kBET rejection rates were used as the final metric (Buttner et al. 2019).

The silhouette coefficient metric measures how similar one sample is to other samples in its own cluster versus how dissimilar it is to samples in other clusters (Rousseeuw 1987). To avoid biased silhouette coefficient results from unbalanced datasets, we computed the average score of the silhouette coefficient to measure the overall batch effect. We randomly subsampled 80% of the original cells and used the top 20 PCs from the batch-corrected gene expression matrix after PCA dimension reduction. The batch_sil function of the kBET package was used to compute the average silhouette coefficient metric. This process was repeated 10 times to ensure the stability of the average silhouette coefficient scores. Finally, the Wilcoxon signed-rank test with Benjamini and Hochberg correction was performed on the resulting metrics to identify if any batch-correction algorithm was statistically significantly better than the others.

However, we found that in some circumstances, the local batch effect could not be measured properly because the metrics above tended to evaluate the global batch-correction performance. Moreover, the quantitative measurements did not test the biological rationale of the corrected gene-correction matrix and downstream analysis. Therefore, we performed inspection at the biological level to examine and analyze the local performance of the batch-correction software and test whether the corrected DGE results were biologically rational.

### Computing time benchmarks

To compare the computation resources used by the four batch-correction methods, we recorded the CPU time on a Linux workstation equipped with 256 GiB memory and 32 2.10 GHz CPU cores. This study demonstrates the performance of four batch-correction methods in processing datasets with two different single-cell sequence technologies.

## Results

### Intratissue performance evaluation intra-tissue

**Fig. 1:**
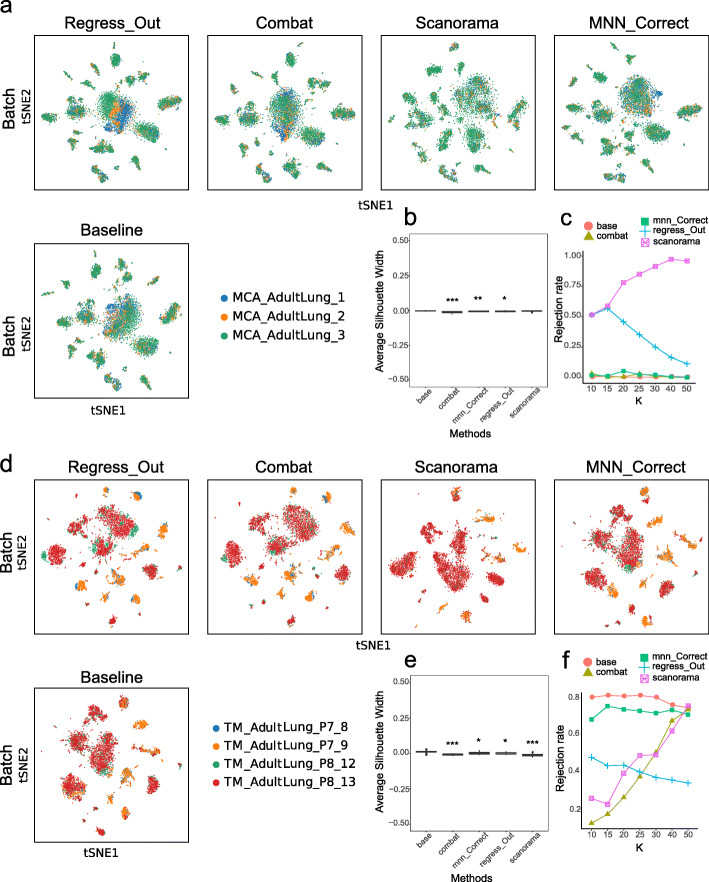
Batch-corrected results for lung data from MCA and TM. **a,** The t-SNE plots present the degree of the batch effect from the MCA lung data (consisting of 3 experimental batches) before correction (baseline) and after correction with 4 methods (Regress_Out, ComBat, Scanorama and MNN_Correct). **b, c,** ASW_batch (boxplot) and the kBET rejection rate (line chart) evaluate the batch-correction effect in the MCA lung data. **d,** The t-SNE plots present the degree of the batch effect from the TM lung data (consisting of 4 batches) before correction (baseline) and after correction using the 4 methods (Regress_Out, ComBat, Scanorama and MNN_Correct). **e, f,** ASW_batch (boxplot) and the kBET rejection rate (line chart) evaluate the batch-correction effect in the TM lung data. *p < 0.05, **p < 0.01, ***p < 0.001; the Wilcoxon signed-rank test with Benjamini and Hochberg correction was performed between each of the four postcorrection groups and the baseline group

To visually demonstrate the effects of the four batch-correction methods, we applied the methods on processed lung data from the MCA and TM datasets. The MCA lung data consist of three experimental batches, which include MCA_AdultLung_1 (2512 cells), MCA_AdultLung_2 (1414 cells) and MCA_AdultLung_3 (3014 cells). To remove the batch effect, we applied the four batch-correction methods and directly integrated the gene expression matrix as a baseline. We noticed that ComBat performed better, in which cells from different experimental batches became well mixed (Fig. 1a). Overlaying the identified cell type information onto the t-SNE plot revealed that the same cell type from different batches was well aligned. For instance, in the baseline result, we observed that epithelial cells and leukocytes mainly composed MCA_AdultLung_3. After the ComBat process, the cells from the three batches identified as one of these two cell types were well integrated (Figure S1a). Further quantified indicators were introduced to evaluate the effectiveness of these four algorithms in removing batch effects. Compared to that of the baseline, the ASW_batch score of ComBat was significantly reduced compared to baseline (*p* < 0.001), while those of MNN_Correct and Regress_Out also decreased (*p* < 0.05), suggesting that these three methods achieved a more uniform cell distribution between batches (Fig. 1b). The ASW_cluster score of Scanorama was significantly different from that of the baseline (*p* < 0.01), indicating that the Scanorama process had a certain influence on the clustering effect (Fig S2a, b). Furthermore, we observed that ComBat and MNN_Correct showed good kBET results, but they were not statistically significant (Fig. 1c).

We performed the same analysis for the TM lung data, which consist of four experimental batches, including TM_AdultLung_P7_8 (462 cells), TM_AdultLung_P7_9 (1286 cells), TM_AdultLung_P8_12 (963 cells) and TM_AdultLung_P8_13 (2789 cells). At baseline, significant batch differences were observed between two of the batches (P7_9 and P8_13), and after applying the four batch-correction methods, the batch effects were all eliminated to some extent (Fig. 1d). In this case, Combat and Scanorama performed best, in which cells from the two batches showed reasonable integration on the t-SNE plot. One of the visible changes was that P8_13 contributed to the majority of the NK cells before the batch-correction process; afterwards, however, all four batch-derived cells were well integrated (Fig. 1d and S1b). The ASW_batch scores of ComBat and Scanorama were significantly lower than that of the baseline (*p* < 0.001), suggesting that these two methods caused the cells from the four batches to mix more uniformly. The scores of MNN_Correct and Regress_Out also improved to some extent over that of the baseline (*p* < 0.05) (Fig. 1e). For the TM lung data, the ASW_cluster scores of ComBat, Regress_Out and MNN_Correct all affected the clustering results (p < 0.001) (Fig S2c, d). The kBET evaluation indexes indicated that ComBat has the lowest rejection rate (*p* < 0.01), and the indexes of Scanorama and Regress_Out were also different from that of the baseline (p < 0.01), indicating that all three methods made particular corrections to the batch effect (Fig. 1f).

### Validation of the intratissue performance

We further selected the MCA_Kidney and TM_Kidney datasets, applied the four algorithms to perform batch correction and evaluated their effects (Fig S3, S4, S5). The MCA_Kidney dataset consists of 3 batches of scRNA-seq data. It is worth noting that 2 batches are from fetal mice and the other batch is from adult mice. Considering the differences between fetal kidney and adult kidney tissues in terms of cell type composition and gene expression patterns, the large differences between the batches in the t-SNE plot are reasonable (Fig S3a). The ASW_batch scores of MNN_Correct and Regress_Out were significantly lower than that of the baseline (*p* < 0.001) (Fig S3b). The ASW_cluster scores of ComBat, Regress_Out and Scanorama changed significantly (*p* < 0.001) (Fig S5a, b). The kBET results indicated that ComBat, Regress_Out and MNN_Correct significantly improved the batch uniformity (p < 0.001) (Fig S3c).

The TM dataset contains data from three batches of adult mouse kidneys. It can be observed from the baseline data that a group of epithelial cells were mainly derived from P7_5, showing a significant batch difference. The integration effect improved after the ComBat and Scanorama processes were run (Fig S3d). The ASW_batch scores of the four algorithms were all significantly lower than that of the baseline (*p* < 0.01) (Fig S3e). The ASW_cluster scores of ComBat, MNN_Correct, and Regress_Out were significantly different from that of the baseline (p < 0.001) (Fig S5c, d). The kBET results showed no significant difference between the four algorithms and the baseline rejection rates (Fig S3f).

### Performance evaluation across different tissues

**Fig. 2:**
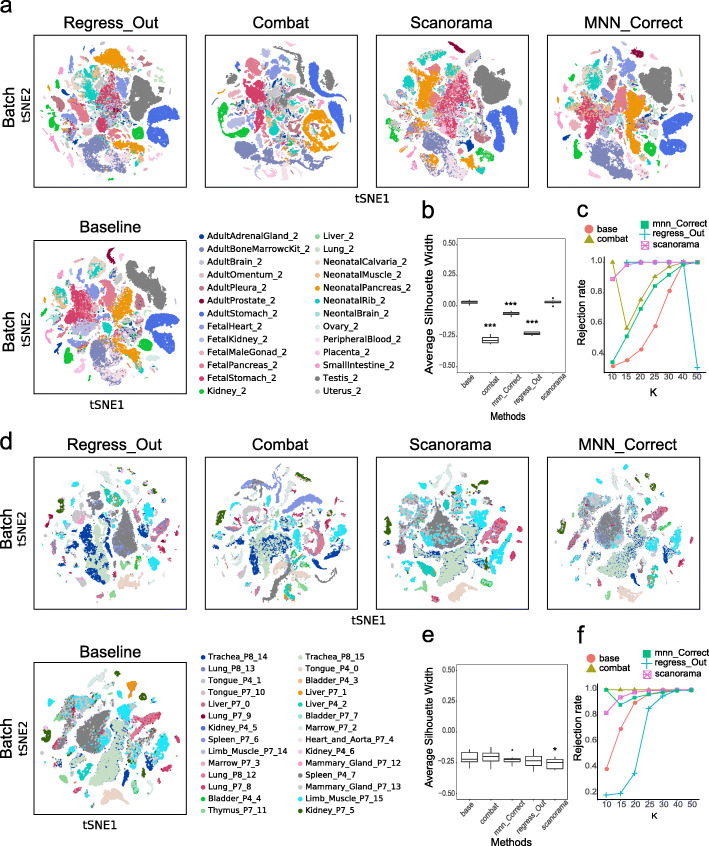
Batch-correction results for multiple tissues from MCA and TM. **a,** The t-SNE plots present the degree of the batch effect from the MCA multitissue data (containing 26 experimental batches of 23 tissues) before correction (baseline) and after correction using four methods (Regress_Out, ComBat, Scanorama and MNN_Correct). **b, c,** ASW_batch (boxplot) and the kBET rejection rate (line chart) evaluate the batch-correction effect in the MCA multitissue data. **d,** The t-SNE plots present the degree of the batch effect from the TM multitissue data (containing 28 experimental batches of 12 tissues) before correction (baseline) and after correction using the four methods (Regress_Out, ComBat, Scanorama and MNN_Correct). **e, f,** ASW_batch (boxplot) and the kBET rejection rate (line chart) evaluate the batch-correction effect in the TM multitissue data. **p < 0.01, ***p < 0.001; the Wilcoxon signed-rank test with Benjamini and Hochberg correction was performed between each of the four postcorrection groups and the baseline group

Furthermore, we evaluated the four batch-correction methods in the processing of data from multiple experimental batches, including the 26 experimental batches of the 23 tissues from the MCA database and the 28 experimental batches of the 12 tissues from the TM database. In both datasets, overlaying tissue information onto the t-SNE plot revealed that these subpopulations corresponded to the different tissues in the baseline results (Fig. 2). Comparing the four methods, Scanorama and Combat performed well, while Regress_Out and MNN_Correct performed somewhat poorly within tissue types. For example, the batch effects of liver tissue from the TM database and of lung tissue from the MCA database could not be eliminated when using Regress_Out and MNN_Correct, respectively. Similarly to the intratissue evaluation, to more objectively compare the batch-correction effects of the four algorithms, we introduced three quantitative indicators: ASW_batch, ASW_cluster, and kBET. First, when processing the MCA dataset, we found that the ASW_batch scores of ComBat, Regress_Out and MNN_Correct were significantly lower than that of the baseline (*p* < 0.001), suggesting that these three methods perform well in improving the uniformity of the mixed cells from the different batches (Fig. 2b). The ASW_cluster scores of all four methods were significantly different from that of the baseline (*p* < 0.01) (Fig S7b), indicating that after integrating multiple batches, these four methods affected the clustering results. One good example is that in the grouping results obtained after running the ComBat process, the cell subgroups in the middle were significantly more chaotic (Fig S7a). There was no statistically significant difference between the four methods and the baseline in terms of the kBET results (Fig. 2c).

The results were different when processing the TM dataset. The ASW_batch score of Scanorama was significantly lower than that of the baseline (*p* < 0.05) (Fig. 2e), while its ASW_cluster score was not different from the baseline score (Fig S7d). It is worth mentioning that the ASW_cluster score measures the degree of aggregation of Louvain clusters, and the effect of the cluster results needs to be further discussed in terms of biological significance. For instance, Scanorama eliminates within-tissue batch effects well within but preserves certain between-tissue batch effects, such as those between marrow and thymus tissue from the TM database. On the t-SNE map, the marrow and thymus are integrated after using Scanorama (Fig. 2d). Combining the results of cell annotation (Fig S6), B cells in the marrow and T cells in the thymus are integrated, possibly due to the similarity in the expression profiles of these immune cells. This may explain the integrated state between marrow and thymus when using Scanorama. As with the MCA database, there was no statistically significant difference between the four methods compared with the baseline in the kBET results for the TM database (Fig. 2f).

In addition, we selected two more datasets containing multiple tissues (TM_P4 with 7823 cells and TM_P7 with 21,383 cells) and evaluated them using the three quantitative indicators (ASW_batch, ASW_cluster and kBET). We discovered that ComBat always performed better than the other methods with these datasets (Fig S8).

During the exploration, we noticed that kBET was insufficiently stable. When faced with different datasets, the results displayed by kBET were highly variable. On the other hand, the two ASW indicators were relatively stable. Therefore, we chose ASW_batch and ASW_cluster as quantitative indicators to evaluate the batch-correction effect.

### Computing time benchmarks

In addition to comparing the batch-correction performance, we also recorded the time used by the four algorithms to process datasets of different sizes. To obtain such datasets, we downsampled the MCA and TM datasets to obtain a total of 9 sets of data containing between ~ 2000 and ~ 140,000 cells, while the number of highly variable genes (HVGs) was controlled in a range from ~ 2000 to ~ 3000 (Table S1). We found that Scanorama and ComBat consumed less time than Regress_Out and MNN_Correct when processing relatively small datasets (< 10,000 cells and < 10 batches) (Fig S9a). When processing a small data set of ~ 2000 cells, all four methods took less than 2 min. For datasets with more than 4000 cells, Regress_Out and MNN_Correct needed up to tens of minutes to complete the process; Scanorama only took approximately 2–6 min, whereas ComBat has the highest efficiency, completing the process in less than 2 min. A similar result was found when processing a larger data set (> 10,000 cells or > 10 batches). The order of the algorithms in terms of the computing times is as follows: MNN_Correct > Regress_Out > Scanorama > Combat (Fig S9b). Note that MNN_Correct took more than 660 min to process fewer than 50,000 cells. In summary, when working with an scRNA-seq dataset with a large number of cells and a large number of batches to be integrated, ComBat, Scanorama and Regress_Out are more recommended if the time cost is the only consideration.

## Discussion

Although the Regress_Out and ComBat algorithms are both based on linear regression, we noticed the superiority of the ComBat method compared to the Regress_Out method in terms of the batch-correction performance and the computing efficiency (Figs. 1, 2, S9). The Regress_Out algorithm uses a general linear model (GLM) to regress out unwanted sources of variation in the expression matrix. The coefficient for each batch block is estimated by fitting the GLM and is then set to zero to remove the corresponding batch effect. The ComBat algorithm uses the same strategy but performs an empirical Bayes (EB) framework to adjust the expression matrix for batch correction. The EB framework is usually designed to shrink gene variances and remove the inferred batch effect by fitting a Bayesian model. ComBat first standardizes all gene expression values across cells and fits the data into a standard distribution Bayesian model. Then, it can infer the batch effects using the estimated model and adjust the gene variances (Johnson et al. 2007a; Leek 2014). Due to the empirical Bayesian shrinkage of the blocking coefficient estimates, the ComBat algorithm is quite robust when processing diverse batches, which is also reflected in our analysis in both the MCA and TM data sets (Figs. 1, 2).

Different from the linear regression used in Regress_Out and ComBat, the MNN_Correct method and Scanorama method search MNNs between batches to adjust the expression matrix. MNNs define the most similar cells across batches in single-cell gene expression analysis. Compared to the linear regression-based methods, MNN-based methods regard less of the predefined or equal population compositions across batches and tend to merge similar cells across batches. For instance, in our performance evaluation across different tissues, we noticed that Scanorama integrated B cells in the marrow and T cells in the thymus due to the similarity in the expression profiles of the immune cells (Fig. 2d, Fig S6).

Moreover, we noticed that the Scanorama method gains a significant speed advantage over MNN_Correct, especially when we perform batch correction in a large data set (Fig S9). MNN_Correct first uses cosine normalization to scale all gene expression values and compute the Euclidean distances to identify mutual nearest neighbors. Then, the differences in the expression values of the identified MNN pairs are calculated to estimate the batch effect. Finally, the batch-correction vector is computed and applied to adjust the expression matrix (Haghverdi et al. 2018). MNNs searching between batches usually spends considerable computing time. Two procedures in Scanorama are implemented to improve the performance of the MNN searches. First, randomized singular value decomposition (SVD) is used to reduce the dimensions of the original gene expression matrix. The SVD compression procedure helps to speed up the MNN search and improve the robustness of the algorithm. Second, unlike the MNN pair search in the MNN_Correct algorithm, Scanorama finds the nearest neighbors among all data sets and creates a panorama using a weighted average of vectors (Hie et al. 2019). Therefore, Scanorama is more insensitive to input order and less vulnerable to overcorrection.

## Conclusions

In this article, we compared and evaluated four Scanpy-based batch-correction methods using representative single-cell transcription datasets. First, we selected a large number of single-cell transcription public datasets, including complex experimental and technological batches. Selected data sets were organized to represent common single-cell data integration scenarios for intratissue and across-tissue batch correction. Our results indicated that, among the four batch-correction methods investigated here, the ComBat method performed the most efficiently and robustly in most of the scenarios we evaluated. The superior batch-correction performance of the ComBat is due to its integrated empirical Bayes (EB) framework. Regardless of the presumption of equal population composition, Scanorama also effectively corrected and integrated the gene expression matrix of diverse batches with relatively reasonable computing resource requirements. We then discussed the performance differences among the evaluated methods at the algorithm level. In conclusion, we recommend employing the ComBat and Scanorama methods to correct batch effects when integrating large single-cell transcriptome datasets.

## Supplementary information

**Additional file 1 : Figure S1.** Identified cell-type information from lung data from the MCA and TM datasets overlaid onto the t-SNE plot. a, The t-SNE plots present the alignment of 14 previously identified cell types in the lung from the MCA dataset before and after using four batch-correction methods. b, The t-SNE plots present the alignment of 12 previously identified cell types in the lung from the TM dataset before and after using the four batch-correction methods.

**Additional file 2 : Figure S2.** Unsupervised clustering results for lung data from the MCA and TM datasets. a, The t-SNE plots visualize the results of the unsupervised clustering of the MCA lung data before and after using four batch-correction methods. b, ASW_cluster (boxplot) measures the degree of aggregation of the Louvain clusters in the MCA lung data. c, The t-SNE plots visualize the results of the unsupervised clustering of the TM lung data before and after using the four batch-correction methods. d, ASW_cluster (boxplot) measures the degree of aggregation of the Louvain clusters in the TM lung data. **p < 0.01, ***p < 0.001; the Wilcoxon signed-rank test with Benjamini and Hochberg correction was performed between each of the four postcorrection groups and the baseline group.

**Additional file 3 : Figure S3.** Batch-corrected results for kidney data from the MCA and TM datasets. a, The t-SNE plots present the degree of the batch effect from the MCA kidney data (consisting of 3 experimental batches) before correction (baseline) and after correction using 4 methods (Regress_Out, ComBat, Scanorama and MNN_Correct). b, c, ASW_batch (boxplot) and the kBET rejection rate (line chart) evaluate the batch-correction effect in the MCA kidney data. d, The t-SNE plots present the degree of the batch effect from the TM kidney data (consisting of 3 batches) before correction (baseline) and after correction using the 4 methods (Regress_Out, ComBat, Scanorama and MNN_Correct). e, f, ASW_batch (boxplot) and the kBET rejection rate (line chart) evaluate the batch-correction effect in the TM kidney data. **p < 0.01, ***p < 0.001; the Wilcoxon signed-rank test with Benjamini and Hochberg correction was performed between each of the four postcorrection groups and the baseline group.

**Additional file 4 : Figure S4.** Identified cell-type information from kidney data from the MCA and TM datasets overlaid onto the t-SNE plot. a, The t-SNE plots present the alignment of 14 previously identified cell types in the kidney from the MCA dataset before and after using four batch-correction methods. b, The t-SNE plots present the alignment of 6 previously identified cell types in kidney from the TM dataset before and after using the four batch-correction methods.

**Additional file 5 : Figure S5.** Unsupervised clustering results for kidney data from the MCA and TM. a, The t-SNE plots visualize the results of unsupervised clustering of the MCA kidney data before and after using four batch-correction methods. b, ASW_cluster (boxplot) measures the degree of aggregation of the Louvain clusters in the MCA kidney data. c, The t-SNE plots visualize the results of unsupervised clustering of the TM kidney data before and after using four batch-correction methods. d, ASW_cluster (boxplot) measures the degree of aggregation of the Louvain clusters in the TM kidney data. ***p < 0.001; the Wilcoxon signed-rank test with Benjamini and Hochberg correction was performed on each of the four postcorrection groups and the baseline group.

**Additional file 6 : Figure S6.** Identified cell-type information from multitissue data from the MCA and TM database overlaid onto the t-SNE plot. a, The t-SNE plots present the alignment of 26 previously identified cell types in multiple tissues from the MCA dataset before and after using four batch-correction methods. b, The t-SNE plots present the alignment of 24 previously identified cell types in multiple tissues from the TM dataset before and after using four batch-correction methods.

**Additional file 7 : Figure S7.** Unsupervised clustering results for multitissue data from the MCA and TM datasets. a, The t-SNE plots visualize the results of the unsupervised clustering of MCA multitissue data before and after using four batch-correction methods. b, ASW_cluster (boxplot) measures the degree of aggregation of the Louvain clusters in the MCA multitissue data. c, The t-SNE plots visualize the results of the unsupervised clustering of the TM multitissue data before and after using four batch-correction methods. d, ASW_cluster (boxplot) measures the degree of aggregation of the Louvain clusters in the TM multitissue data. **p < 0.01, ***p < 0.001; the Wilcoxon signed-rank test with Benjamini and Hochberg correction was performed between each of the four postcorrection groups and the baseline group.

**Additional file 8 : Figure S8.** Quantitative indicators evaluate the batch-correction results from the TM_P4 and TM_P7 datasets. a, ASW_batch (boxplot), ASW_cluster (boxplot) and the kBET rejection rate (line chart) evaluate the batch-correction effect in the TM_P4 data. b, ASW_batch (boxplot), ASW_cluster (boxplot) and kBET rejection rate (line chart) evaluate the batch-correction effect in the TM_P7 data. **p < 0.01, ***p < 0.001; the Wilcoxon signed-rank test with Benjamini and Hochberg correction was performed between each of the four postcorrection groups and the baseline group.

**Additional file 9 : Figure S9.** Computing time costs of the 4 batch-correction methods in processing 9 datasets. a, A line chart presents the computing time costs of the 4 batch-correction methods in 4 small datasets (< 10,000 cells and < 10 batches). b, A line chart presents the computing time costs of the 4 batch-correction methods in 5 large datasets (> 10,000 cells or > 10 batches).

**Additional file 10 : Table S1.** Computing time of the 4 batch-correction methods under different conditions. Record of the computing time of the 4 batch-correction methods (Regress_Out, ComBat, Scanorama and MNN_Correct) in processing 9 datasets independently.

## Data Availability

The code used to reproduce the analysis is available from the following GitHub repository: https://github.com/JiaqiLiZju/Comparison_batch_remove_softwares.

## Abbreviations

scRNA-seq: single-cell RNA sequencing
MNNs: mutual nearest neighbors
SVD: singular value decomposition
MCA: Mouse Cell Atlas
TM: Tabula Muris

## References

[CR1] Buttner M, Miao Z, Wolf FA, Teichmann SA, Theis FJ (2019). A test metric for assessing single-cell RNA-seq batch correction. Nat Methods.

[CR2] Gierahn TM, Wadsworth MH, Hughes TK, Bryson BD, Butler A, Satija R, Fortune S, Love JC, Shalek AK (2017). Seq-well: portable, low-cost RNA sequencing of single cells at high throughput. Nat Methods.

[CR3] Haghverdi L, Lun ATL, Morgan MD, Marioni JC (2018). Batch effects in single-cell RNA-sequencing data are corrected by matching mutual nearest neighbors. Nat Biotechnol.

[CR4] Han X, Wang R, Zhou Y, Fei L, Sun H, Lai S, Saadatpour A, Zhou Z, Chen H, Ye F (2018). Mapping the mouse cell atlas by microwell-Seq. Cell.

[CR5] Hie B, Bryson B, Berger B (2019). Efficient integration of heterogeneous single-cell transcriptomes using Scanorama. Nat Biotechnol.

[CR6] Hwang B, Lee JH, Bang D (2018). Single-cell RNA sequencing technologies and bioinformatics pipelines. Exp Mol Med.

[CR7] Johnson WE, Li C, Rabinovic A (2007). Adjusting batch effects in microarray expression data using empirical Bayes methods. Biostatistics.

[CR8] Klein AM, Mazutis L, Akartuna I, Tallapragada N, Veres A, Li V, Peshkin L, Weitz DA, Kirschner MW (2015). Droplet barcoding for single-cell transcriptomics applied to embryonic stem cells. Cell.

[CR9] Leek JT (2014). svaseq:removing batch effects and other unwanted noise from sequencing data. Nucleic Acids Res.

[CR10] Macosko EZ, Basu A, Satija R, Nemesh J, Shekhar K, Goldman M, Tirosh I, Bialas AR, Kamitaki N, Martersteck EM (2015). Highly parallel genome-wide expression profiling of individual cells using Nanoliter droplets. Cell.

[CR11] Rousseeuw PJ (1987). Silhouettes: a graphical aid to the interpretation and validation of cluster analysis. J Comput Appl Math.

[CR12] Shalek AK, Satija R, Shuga J, Trombetta JJ, Gennert D, Lu D, Chen P, Gertner RS, Gaublomme JT, Yosef N (2014). Single-cell RNA-seq reveals dynamic paracrine control of cellular variation. Nature.

[CR13] Stuart T, Butler A, Hoffman P, Hafemeister C, Papalexi E, Mauck WM, Hao Y, Stoeckius M, Smibert P, Satija R (2019). Comprehensive integration of single-cell data. Cell.

[CR14] Stuart T, Satija R (2019). Integrative single-cell analysis. Nat Rev Genet.

[CR15] Tabula Muris C, Overall c, Logistical c, Organ c, processing, Library p, sequencing, computational data a, Cell type a, Writing g, et al: Single-cell transcriptomics of 20 mouse organs creates a Tabula Muris**.** Nature 2018, 562**:**367–372. 10.1038/s41586-018-0590-4 PMC6642641 30283141

[CR16] Tung PY, Blischak JD, Hsiao CJ, Knowles DA, Burnett JE, Pritchard JK, Gilad Y (2017). Batch effects and the effective design of single-cell gene expression studies. Sci Rep.

[CR17] Wang T, Johnson TS, Shao W, Lu Z, Helm BR, Zhang J, Huang K (2019). BERMUDA: a novel deep transfer learning method for single-cell RNA sequencing batch correction reveals hidden high-resolution cellular subtypes. Genome Biol.

[CR18] Wolf FA, Angerer P, Theis FJ (2018). SCANPY: large-scale single-cell gene expression data analysis. Genome Biol.

[CR19] Zeng T, Dai H (2019). Single-cell RNA sequencing-based computational analysis to describe disease heterogeneity. Front Genet.

