# Supplementary figures and images for: Comparison of Scanpy-based algorithms to remove the batch effect from single-cell RNA-seq data
Source: Cell Regen. 2020 Jul 6;9:10. doi: 10.1186/s13619-020-00041-9 (PMC7338326; doi:10.1186/s13619-020-00041-9)

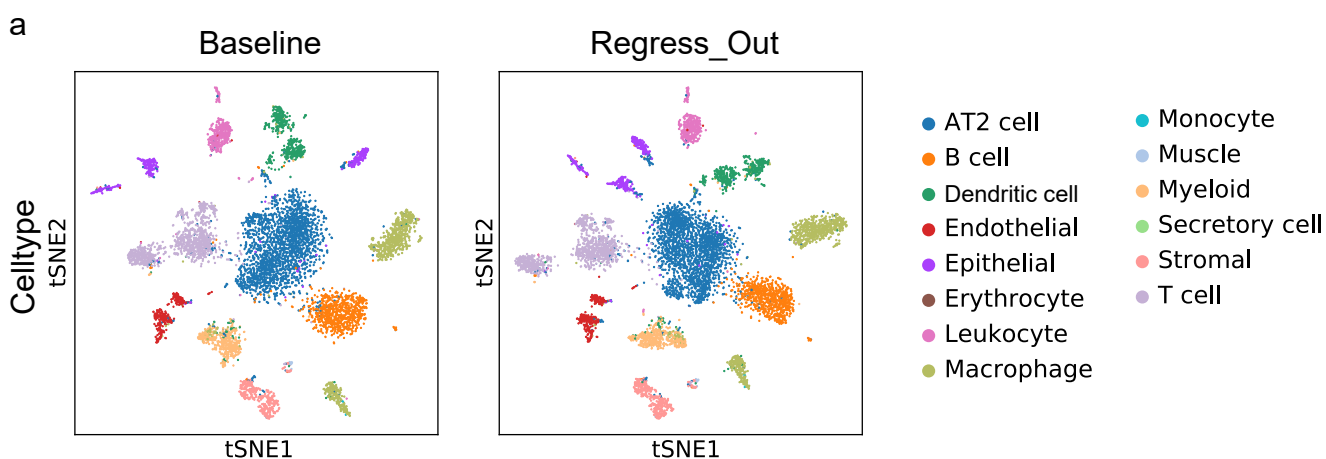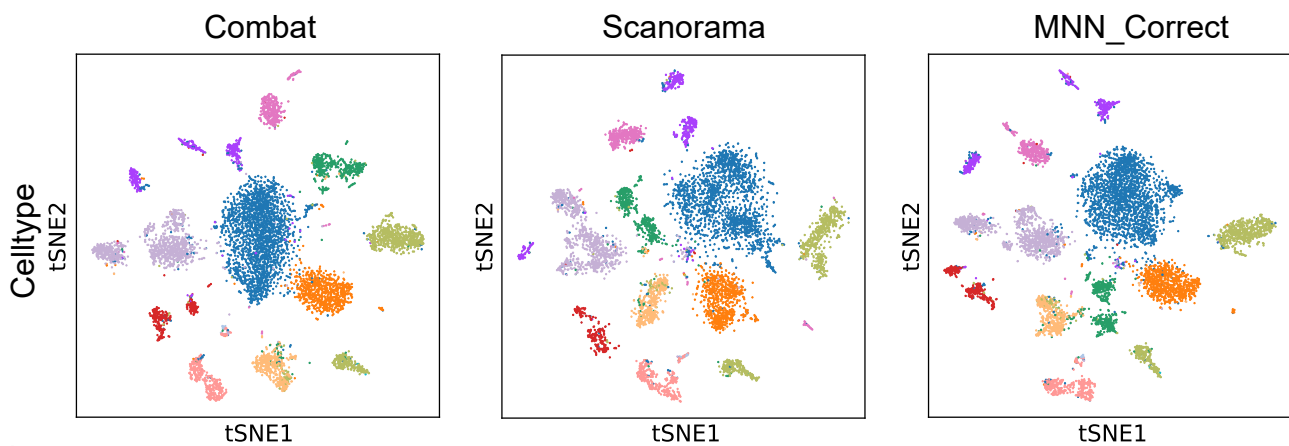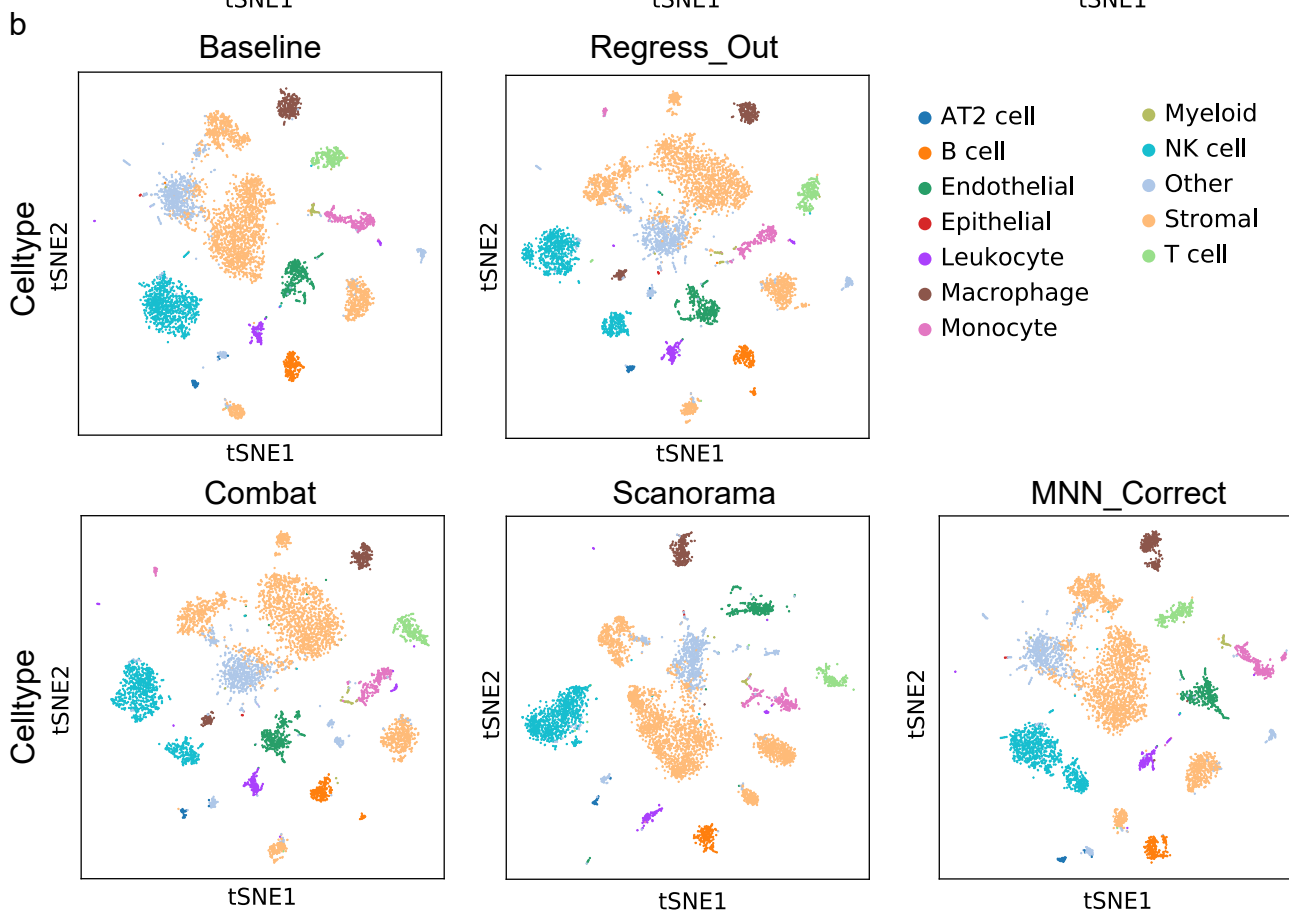

Supplement: Supplementary file 1 — Additional file 1 : Figure S1. Identified cell-type information from lung data from the MCA and TM datasets overlaid onto the t-SNE plot. a, The t-SNE plots present the alignment of 14 previously identified cell types in the lung from the MCA dataset before and after using four batch-correction methods. b, The t-SNE plots present the alignment of 12 previously identified cell types in the lung from the TM dataset before and after using the four batch-correction methods. [file 13619_2020_41_MOESM1_ESM.pdf]

a

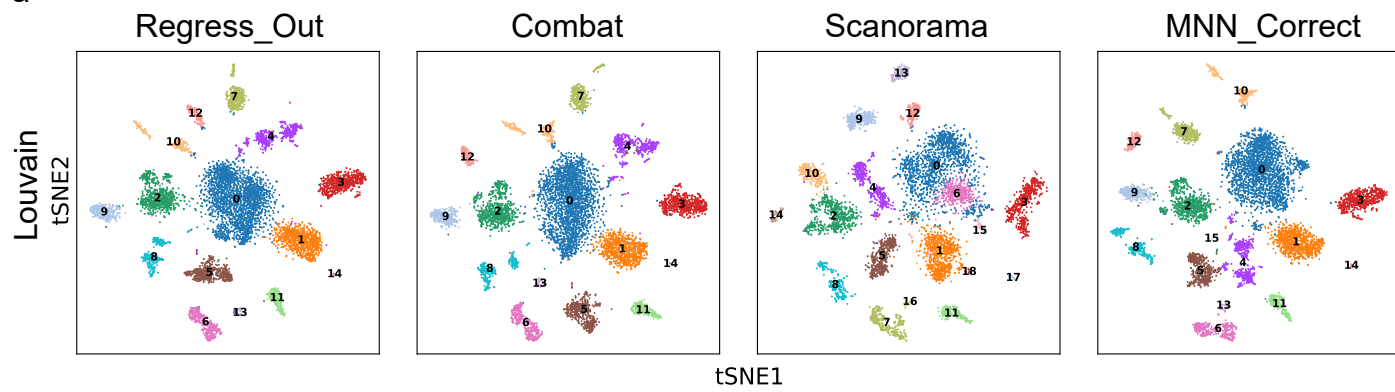

b

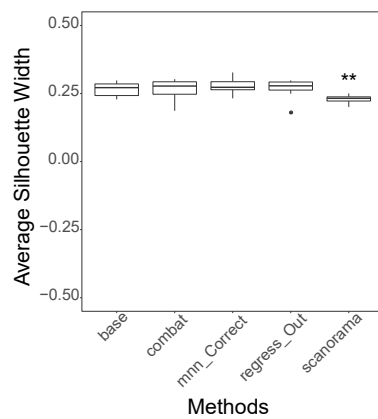

c

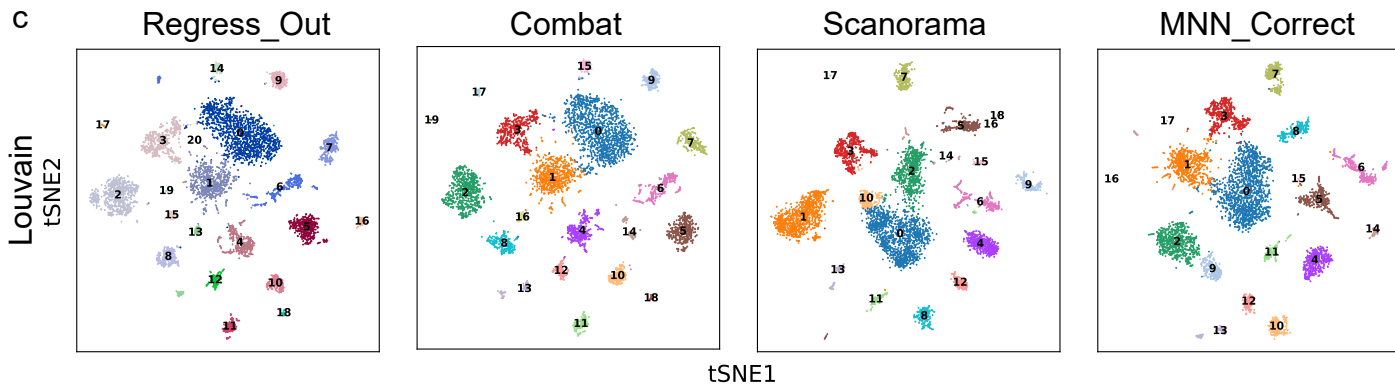

d

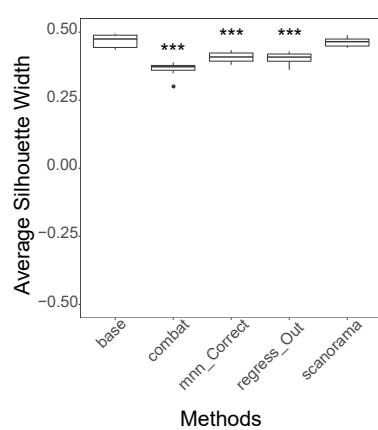

Supplement: Supplementary file 2 — Additional file 2 : Figure S2. Unsupervised clustering results for lung data from the MCA and TM datasets. a, The t-SNE plots visualize the results of the unsupervised clustering of the MCA lung data before and after using four batch-correction methods. b, ASW_cluster (boxplot) measures the degree of aggregation of the Louvain clusters in the MCA lung data. c, The t-SNE plots visualize the results of the unsupervised clustering of the TM lung data before and after using the four batch-correction methods. d, ASW_cluster (boxplot) measures the degree of aggregation of the Louvain clusters in the TM lung data. **p < 0.01, ***p < 0.001; the Wilcoxon signed-rank test with Benjamini and Hochberg correction was performed between each of the four postcorrection groups and the baseline group. [file 13619_2020_41_MOESM2_ESM.pdf]

a

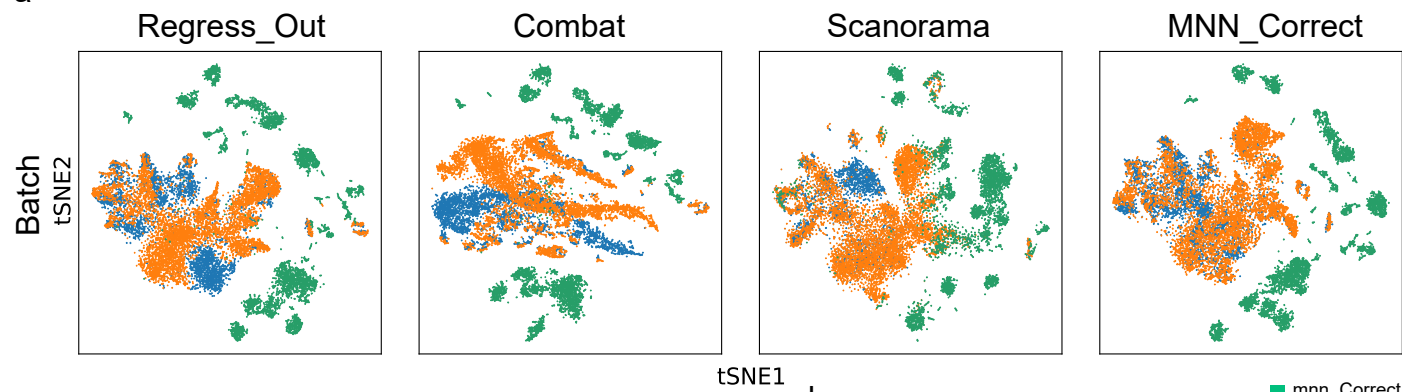

b

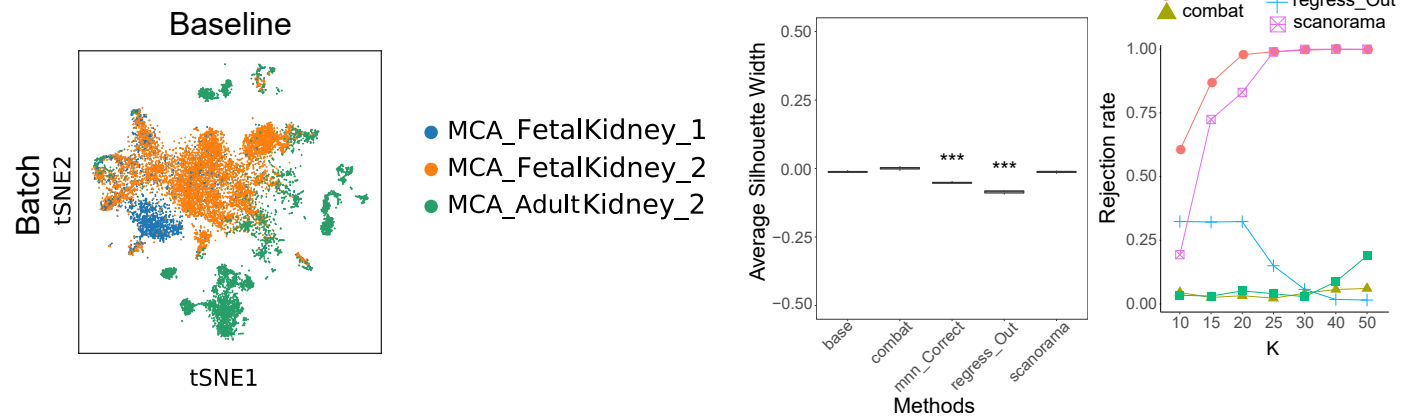

d

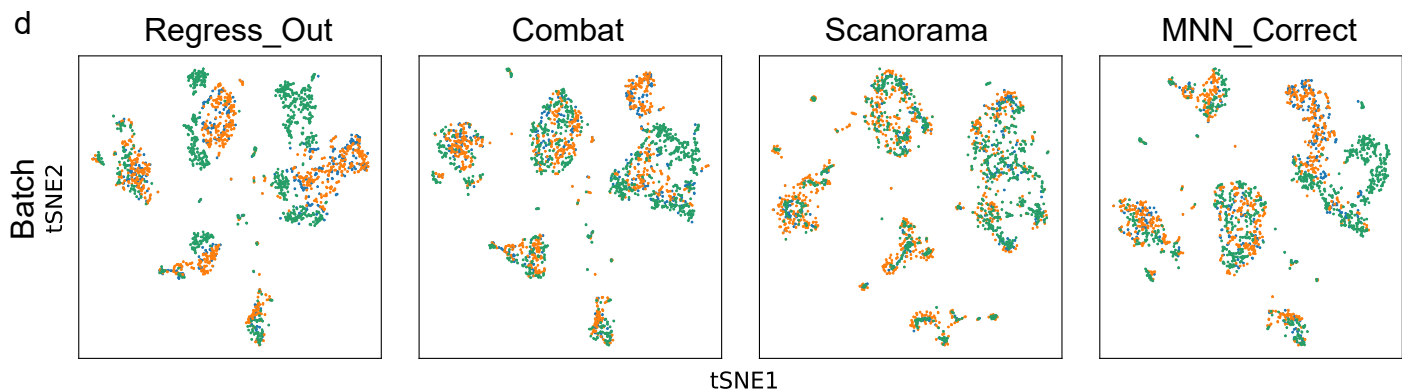

e

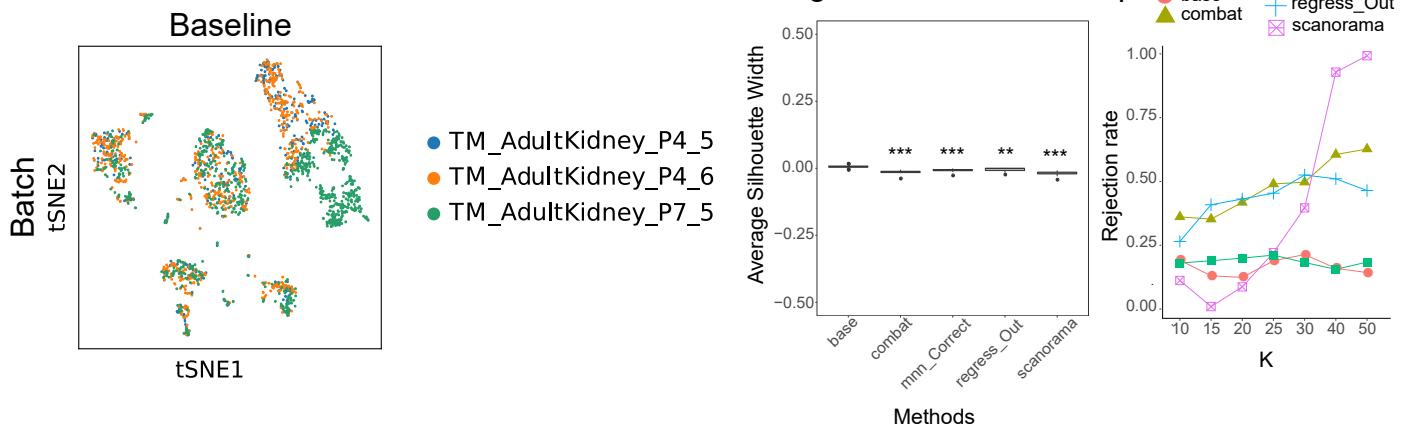

Supplement: Supplementary file 3 — Additional file 3 : Figure S3. Batch-corrected results for kidney data from the MCA and TM datasets. a, The t-SNE plots present the degree of the batch effect from the MCA kidney data (consisting of 3 experimental batches) before correction (baseline) and after correction using 4 methods (Regress_Out, ComBat, Scanorama and MNN_Correct). b, c, ASW_batch (boxplot) and the kBET rejection rate (line chart) evaluate the batch-correction effect in the MCA kidney data. d, The t-SNE plots present the degree of the batch effect from the TM kidney data (consisting of 3 batches) before correction (baseline) and after correction using the 4 methods (Regress_Out, ComBat, Scanorama and MNN_Correct). e, f, ASW_batch (boxplot) and the kBET rejection rate (line chart) evaluate the batch-correction effect in the TM kidney data. **p < 0.01, ***p < 0.001; the Wilcoxon signed-rank test with Benjamini and Hochberg correction was performed between each of the four postcorrection groups and the baseline group. [file 13619_2020_41_MOESM3_ESM.pdf]

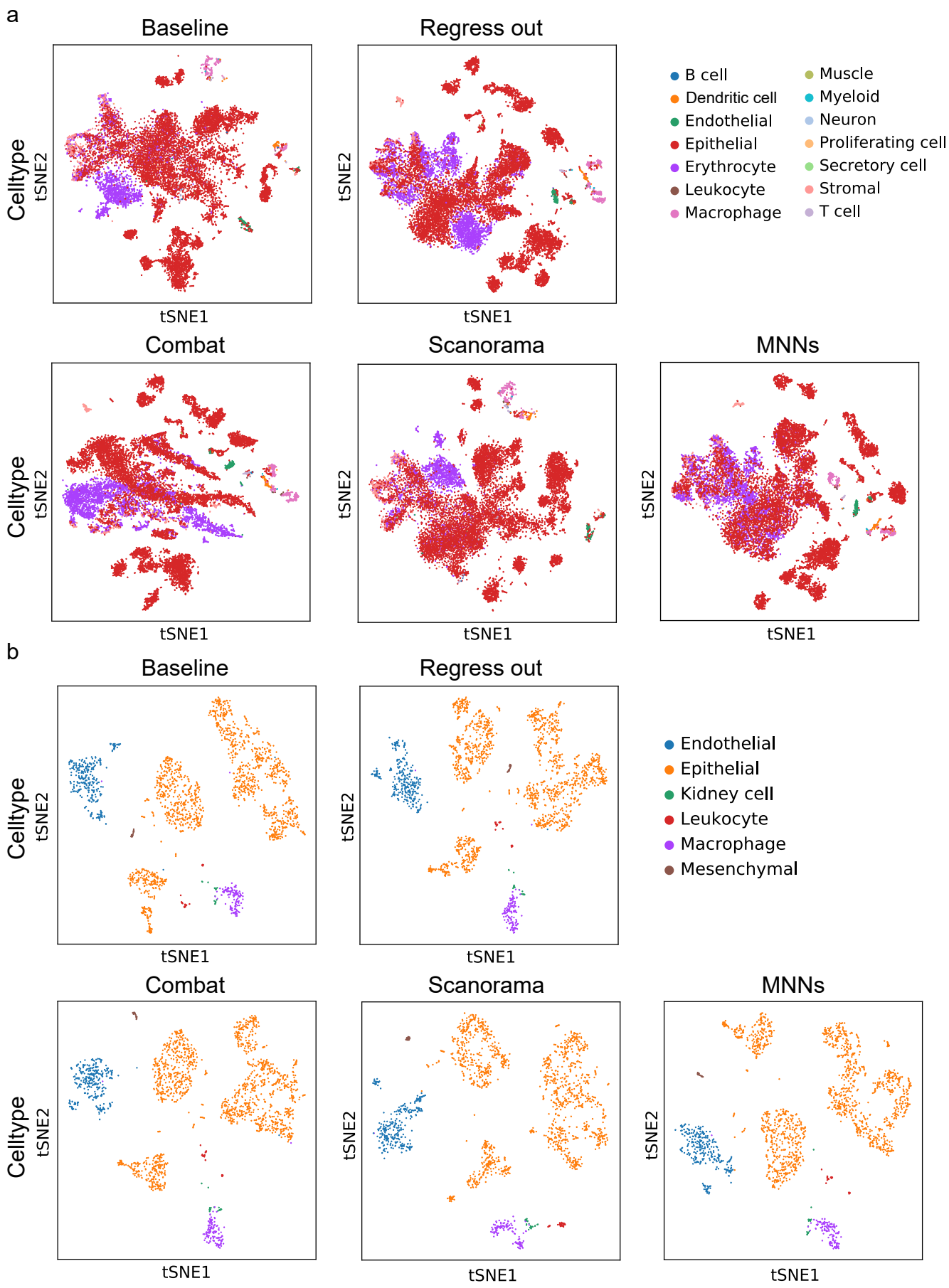

Supplement: Supplementary file 4 — Additional file 4 : Figure S4. Identified cell-type information from kidney data from the MCA and TM datasets overlaid onto the t-SNE plot. a, The t-SNE plots present the alignment of 14 previously identified cell types in the kidney from the MCA dataset before and after using four batch-correction methods. b, The t-SNE plots present the alignment of 6 previously identified cell types in kidney from the TM dataset before and after using the four batch-correction methods. [file 13619_2020_41_MOESM4_ESM.pdf]

a

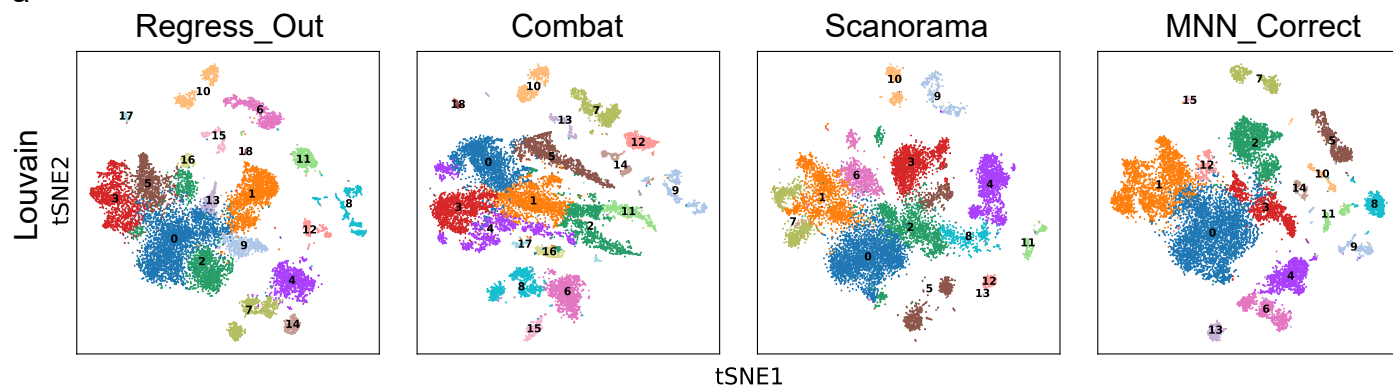

b

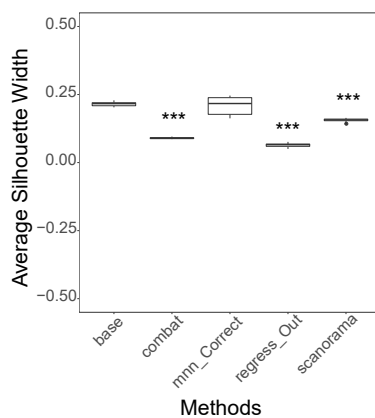

c

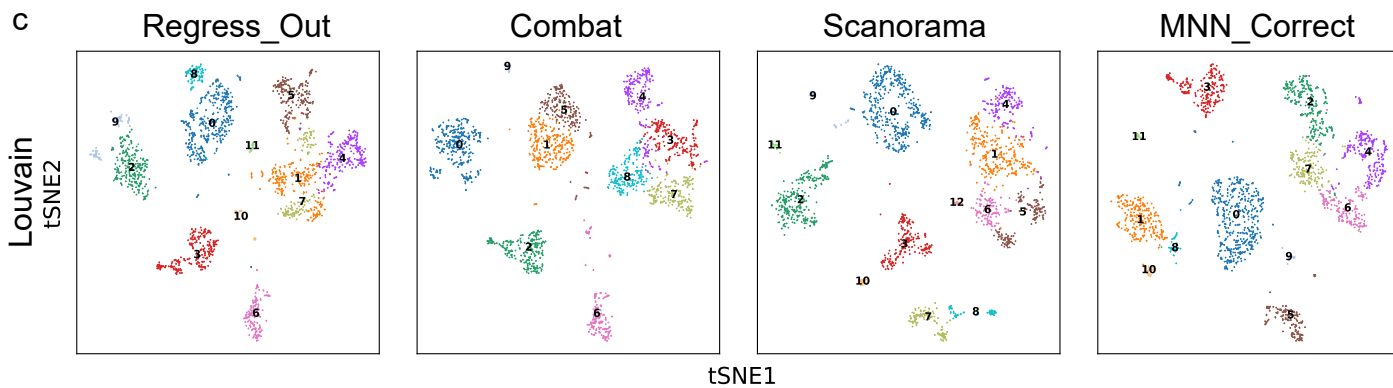

d

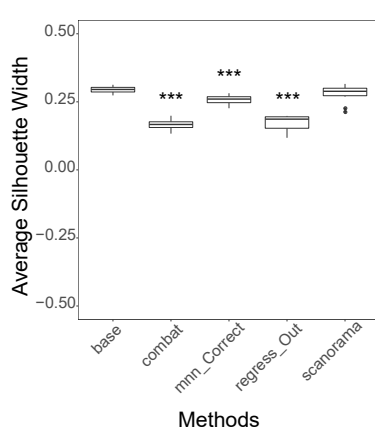

Supplement: Supplementary file 5 — Additional file 5 : Figure S5. Unsupervised clustering results for kidney data from the MCA and TM. a, The t-SNE plots visualize the results of unsupervised clustering of the MCA kidney data before and after using four batch-correction methods. b, ASW_cluster (boxplot) measures the degree of aggregation of the Louvain clusters in the MCA kidney data. c, The t-SNE plots visualize the results of unsupervised clustering of the TM kidney data before and after using four batch-correction methods. d, ASW_cluster (boxplot) measures the degree of aggregation of the Louvain clusters in the TM kidney data. ***p < 0.001; the Wilcoxon signed-rank test with Benjamini and Hochberg correction was performed on each of the four postcorrection groups and the baseline group. [file 13619_2020_41_MOESM5_ESM.pdf]

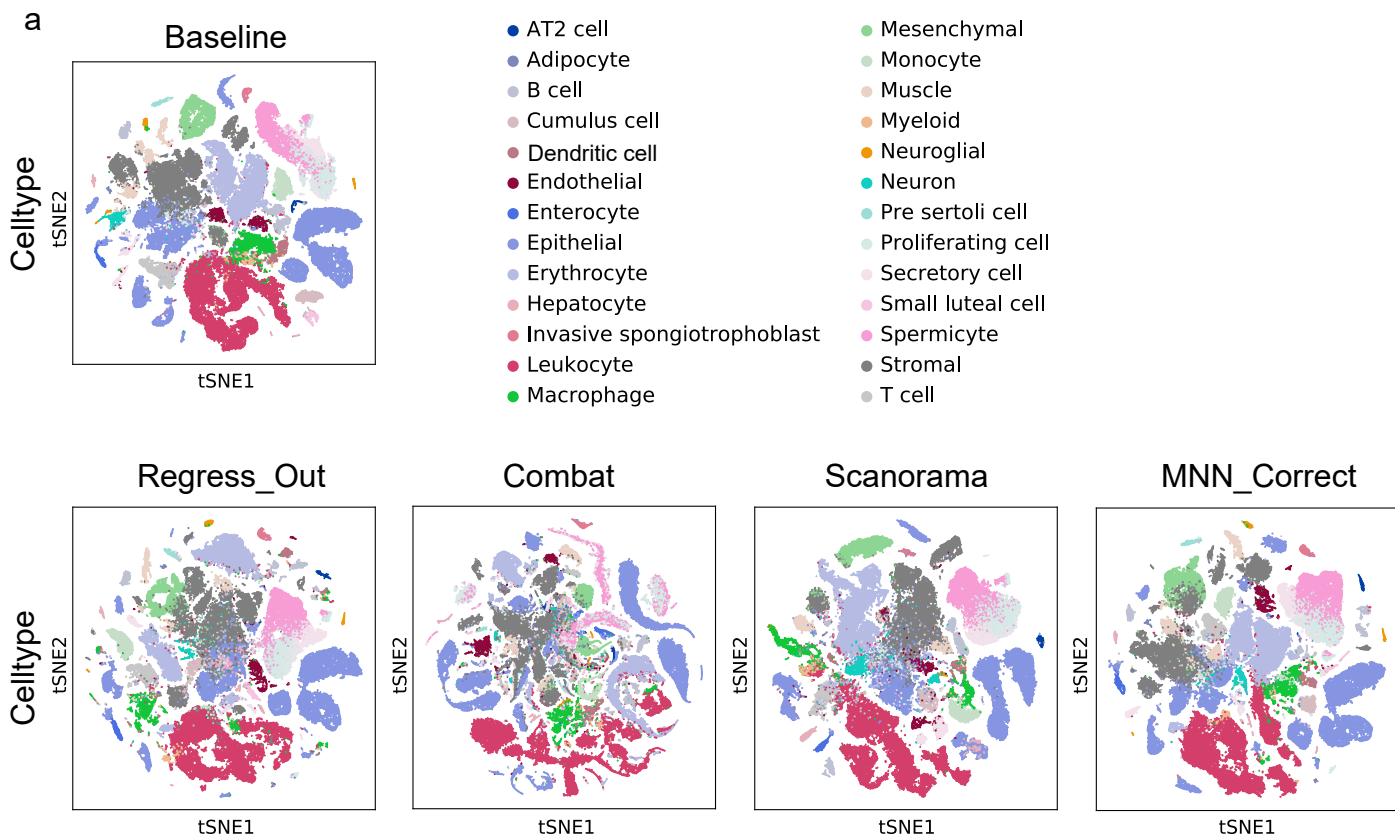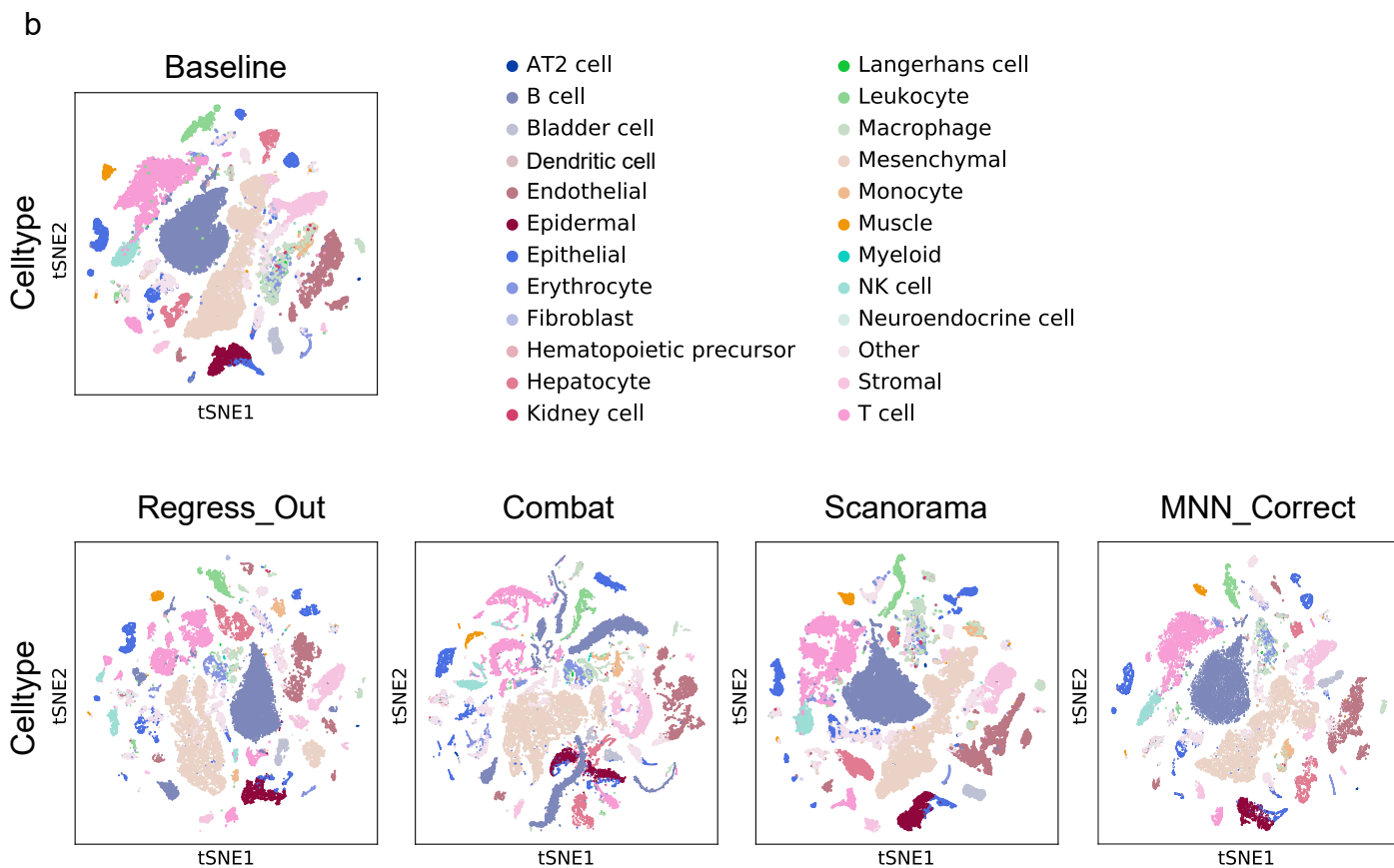

Supplement: Supplementary file 6 — Additional file 6 : Figure S6. Identified cell-type information from multitissue data from the MCA and TM database overlaid onto the t-SNE plot. a, The t-SNE plots present the alignment of 26 previously identified cell types in multiple tissues from the MCA dataset before and after using four batch-correction methods. b, The t-SNE plots present the alignment of 24 previously identified cell types in multiple tissues from the TM dataset before and after using four batch-correction methods. [file 13619_2020_41_MOESM6_ESM.pdf]

a

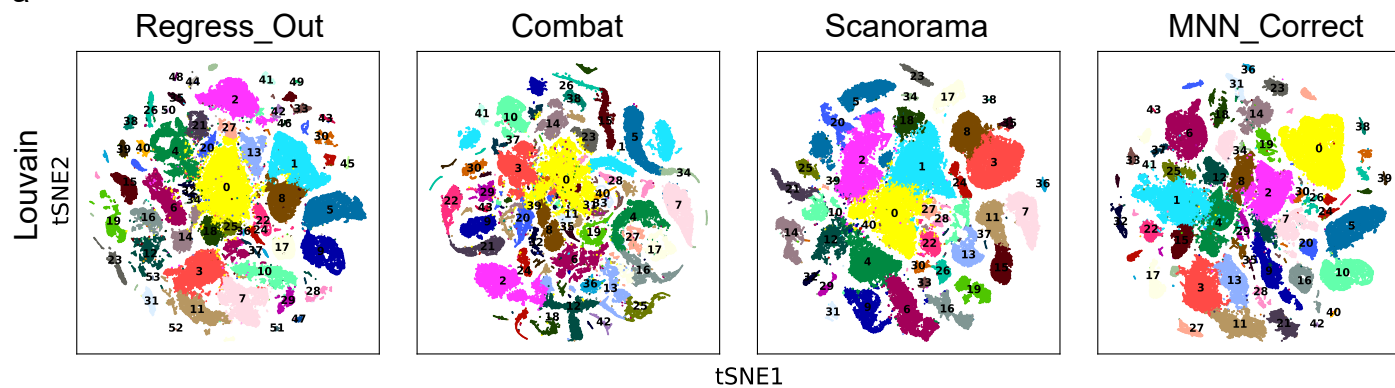

b

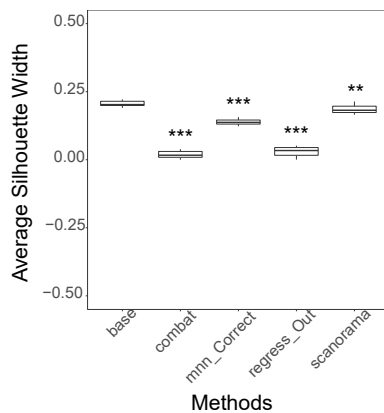

c

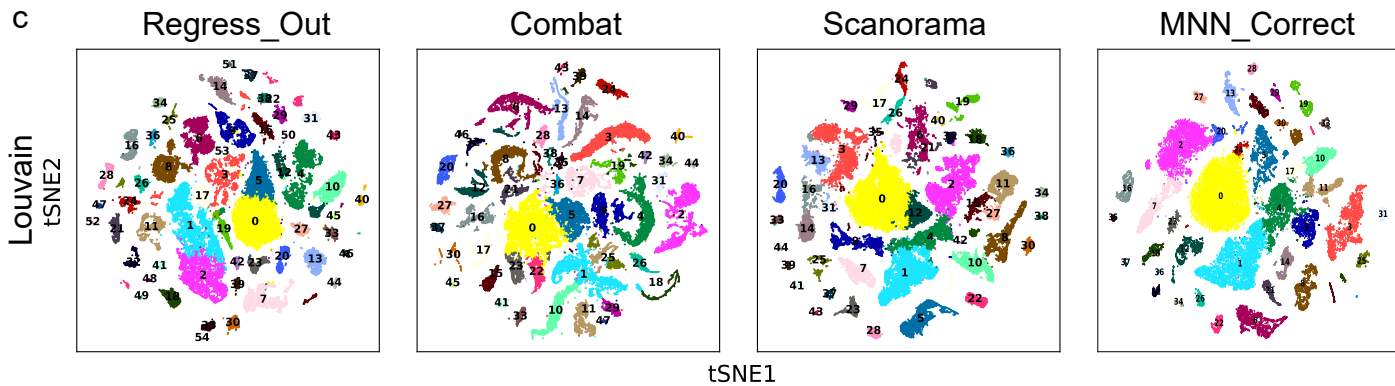

d

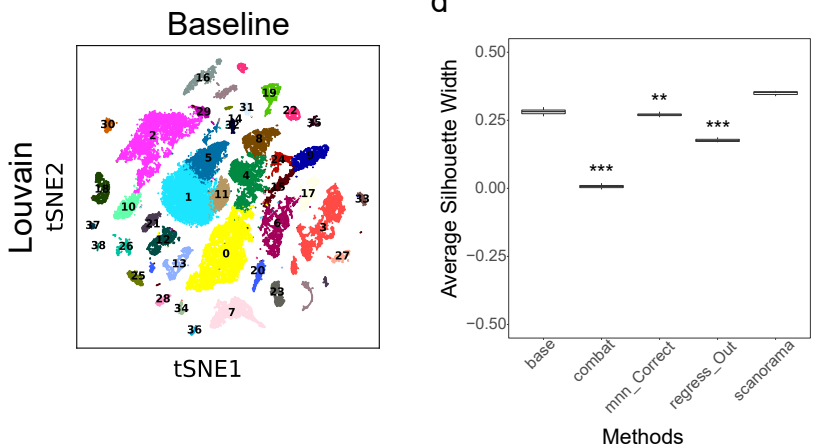

Supplement: Supplementary file 7 — Additional file 7 : Figure S7. Unsupervised clustering results for multitissue data from the MCA and TM datasets. a, The t-SNE plots visualize the results of the unsupervised clustering of MCA multitissue data before and after using four batch-correction methods. b, ASW_cluster (boxplot) measures the degree of aggregation of the Louvain clusters in the MCA multitissue data. c, The t-SNE plots visualize the results of the unsupervised clustering of the TM multitissue data before and after using four batch-correction methods. d, ASW_cluster (boxplot) measures the degree of aggregation of the Louvain clusters in the TM multitissue data. **p < 0.01, ***p < 0.001; the Wilcoxon signed-rank test with Benjamini and Hochberg correction was performed between each of the four postcorrection groups and the baseline group. [file 13619_2020_41_MOESM7_ESM.pdf]

a

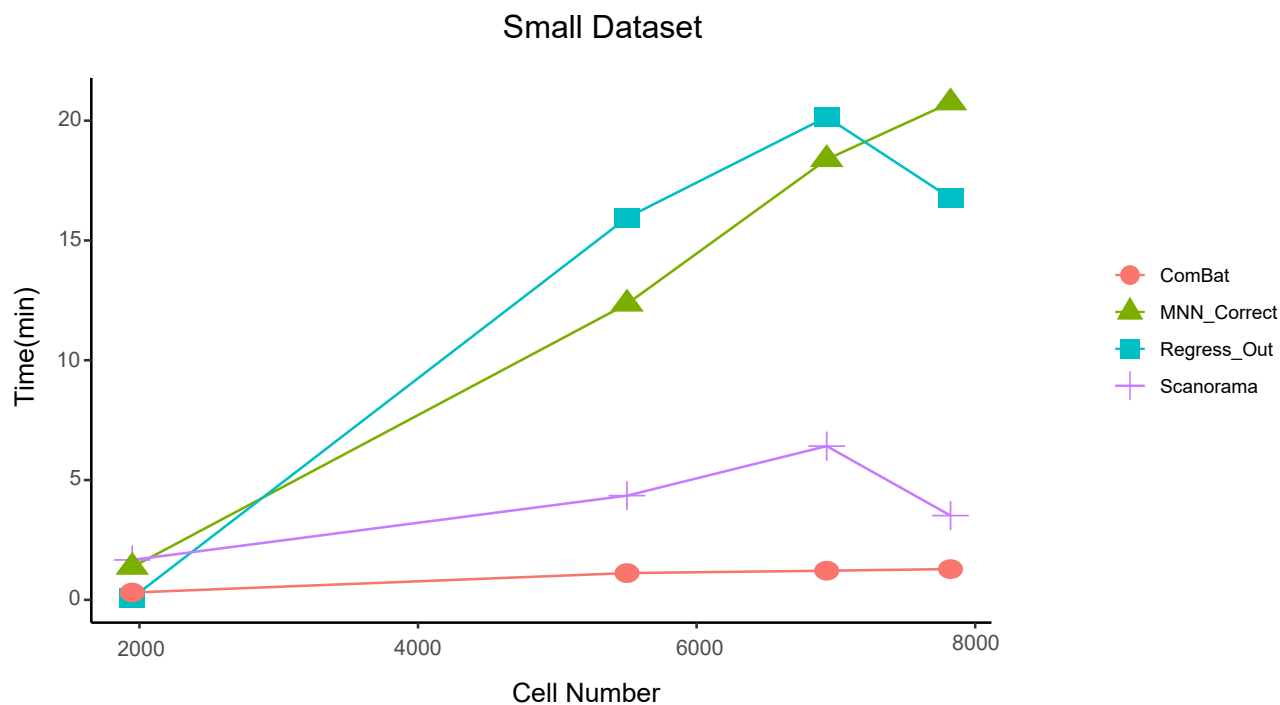

b

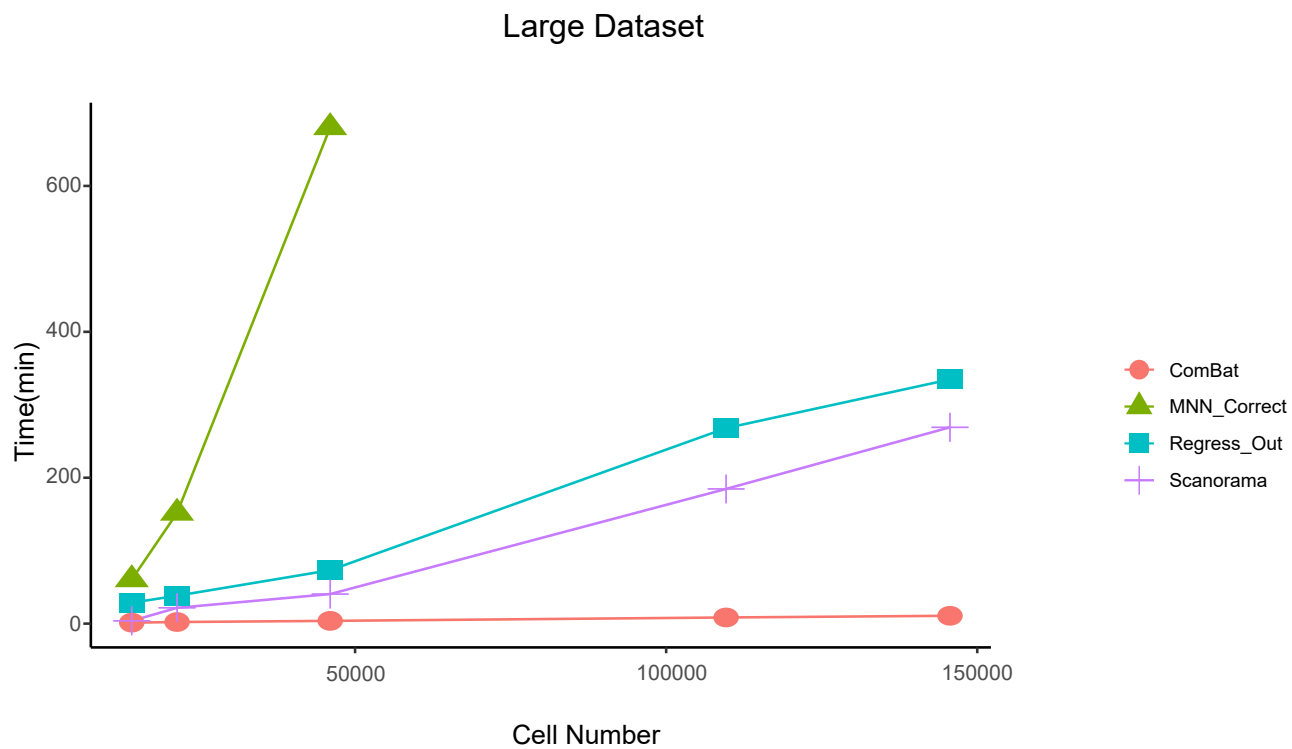

Supplement: Supplementary file 9 — Additional file 9 : Figure S9. Computing time costs of the 4 batch-correction methods in processing 9 datasets. a, A line chart presents the computing time costs of the 4 batch-correction methods in 4 small datasets (< 10,000 cells and < 10 batches). b, A line chart presents the computing time costs of the 4 batch-correction methods in 5 large datasets (> 10,000 cells or > 10 batches). [file 13619_2020_41_MOESM9_ESM.pdf]
