# Supplementary material for: Comparison of Scanpy-based algorithms to remove the batch effect from single-cell RNA-seq data
Source: Cell Regen. 2020 Jul 6;9:10. doi: 10.1186/s13619-020-00041-9 (PMC7338326; doi:10.1186/s13619-020-00041-9)
Supplement: Supplementary file 8 — Additional file 8 : Figure S8. Quantitative indicators evaluate the batch-correction results from the TM_P4 and TM_P7 datasets. a, ASW_batch (boxplot), ASW_cluster (boxplot) and the kBET rejection rate (line chart) evaluate the batch-correction effect in the TM_P4 data. b, ASW_batch (boxplot), ASW_cluster (boxplot) and kBET rejection rate (line chart) evaluate the batch-correction effect in the TM_P7 data. **p < 0.01, ***p < 0.001; the Wilcoxon signed-rank test with Benjamini and Hochberg correction was performed between each of the four postcorrection groups and the baseline group. [file 13619_2020_41_MOESM8_ESM.pdf]

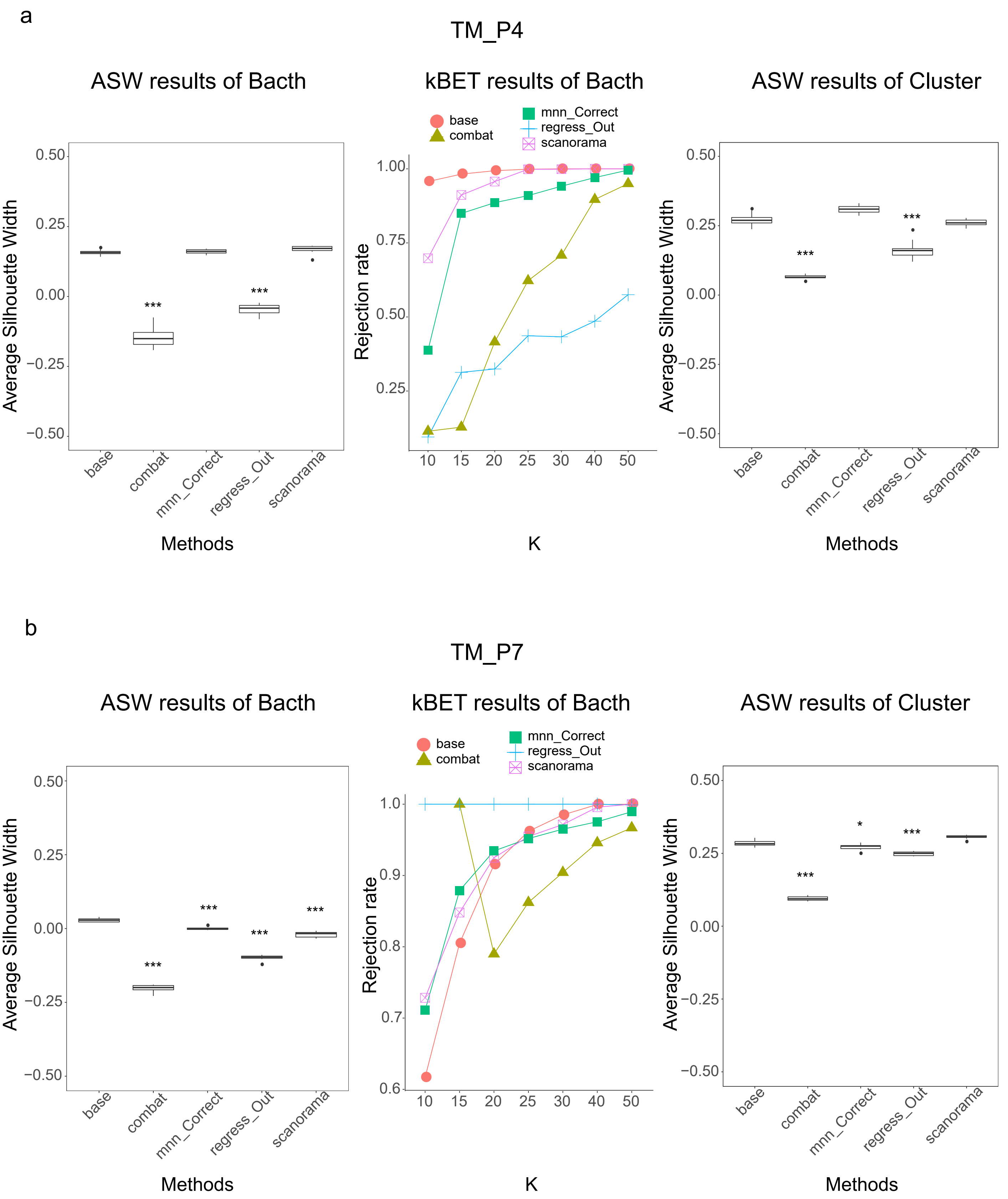
a
TM_P4
ASW results of Bacth
Average Silhouette Width
0.50
0.25
0.00
−0.25
−0.50
***
***
base
combat
mnn_Correct
regress_Out
scanorama
Methods
kBET results of Bacth
base
combat
mnn_Correct
regress_Out
scanorama
Rejection rate
1.00
0.75
0.50
0.25
10
15
20
25
30
40
50
K
ASW results of Cluster
Average Silhouette Width
0.50
0.25
0.00
−0.25
−0.50
***
***
base
combat
mnn_Correct
regress_Out
scanorama
Methods
b
TM_P7
ASW results of Bacth
Average Silhouette Width
0.50
0.25
0.00
−0.25
−0.50
***
***
***
***
base
combat
mnn_Correct
regress_Out
scanorama
Methods
kBET results of Bacth
base
combat
mnn_Correct
regress_Out
scanorama
Rejection rate
1.0
0.9
0.8
0.7
0.6
10
15
20
25
30
40
50
K
ASW results of Cluster
Average Silhouette Width
0.50
0.25
0.00
−0.25
−0.50
***
*
***
base
combat
mnn_Correct
regress_Out
scanorama
Methods
